# Application of Artificial Intelligence and Machine Learning in Arrhythmia Detection and Pacemaker Data Analysis

**DOI:** 10.14740/cr2273

**Published:** 2026-08-31

**Authors:** Lu Huan Shen, Ke Qiang Xu, Peng Fei Xia, Jian Chen

**Affiliations:** a Department of Cardiology, Lanxi People’s Hospital, Lanxi, Zhejiang 321100, China; Hefei, Anhui 230000, China

**Keywords:** Artificial intelligence, Machine learning, Deep learning, Arrhythmia, Pacemaker, Remote monitoring, Clinical decision support

## Abstract

Driven by rapid advancements in computational power and algorithmic innovations, artificial intelligence (AI) and machine learning (ML) technologies have demonstrated significant potential in the field of cardiovascular medicine. This systematic review evaluates the applications of AI/ML in two key areas: arrhythmia detection and pacemaker data analytics. It examines the evolution of these technologies from traditional ML to deep learning, assessing their effectiveness in enhancing diagnostic accuracy, predicting clinical outcomes, and optimizing treatment strategies. Notably, this review supplements the practical application status of AI-augmented algorithms in mainstream clinical electrocardiogram (ECG) reading systems (represented by GE and Siemens platforms) in real-world clinical scenarios and systematically summarizes the latest research progress of AI-based intelligent analysis targeting pacemaker spike signals, which are easily ignored by conventional detection methods. The article also addresses current challenges, including technical limitations, barriers to clinical implementation, and ethical and regulatory considerations. Although issues such as data standardization, model interpretability, and external validation remain, AI/ML technologies already show transformative potential in arrhythmia management and pacemaker data utilization and are expected to play an increasingly central role in the future of personalized cardiology.

## Introduction

Cardiac arrhythmias represent a major global public health challenge, associated with significant morbidity and mortality. According to estimates, atrial fibrillation alone affects over 33 million people worldwide and is associated with a five-fold increased risk of stroke [1]. Early and accurate detection of arrhythmias is crucial for effective management and improved clinical outcomes. Traditionally, the diagnosis of arrhythmias has relied heavily on manual interpretation of electrocardiograms (ECGs) by specialized physicians, a process that is not only time-consuming but also influenced by the clinician’s experience and subjective judgment [2]. In current real-world clinical practice in the United States and most regions, routine ECG interpretation adopts the mode of “automated system preliminary reading + physician manual review.” Mainstream commercial ECG reading systems developed by GE and Siemens are widely embedded in clinical ECG machines and hospital central computing systems, serving as the primary tool for batch ECG preliminary screening. However, these dominant clinical ECG systems still rely on traditional rule-based algorithms, and built-in full-process AI-augmented reading functions have not been widely popularized and applied in routine clinical workflows, resulting in inherent accuracy bottlenecks in automated ECG analysis.

Meanwhile, the use of implantable cardiac electronic devices, such as pacemakers and defibrillators, has become increasingly widespread. These devices not only deliver therapies but also continuously record vast amounts of electrophysiological data [3]. In particular, pacemaker spike signals contained in ECG recordings of patients with implantable pacing devices carry rich clinical information, including pacing device operating status, myocardial pacing capture effect, lead stability, and early abnormal device signals. Conventional ECG and pacemaker data analysis methods often ignore the micro-features of pacemaker spikes or fail to distinguish valid pacing spikes from environmental noise artifacts, leading to underutilization of this key clinical information and missed diagnosis of early pacing abnormalities and arrhythmia precursors. However, conventional data analysis methods are inadequate for fully extracting clinical insights from these complex datasets, leaving much of the potentially useful information underutilized [4]. This gap highlights the urgent need for more advanced analytical tools.

In recent years, artificial intelligence (AI) and machine learning (ML) have led to breakthrough advancements in medical applications [5]. These computational techniques can automatically learn patterns and features from large-scale, complex datasets, offering new opportunities for arrhythmia detection and cardiac device data analysis. Research by Goldberger et al has shown that deep learning algorithms can perform at or even exceed cardiologist-level accuracy in detecting certain types of arrhythmias [6]. Similarly, Tison et al demonstrated that ML models can identify atrial fibrillation from consumer-grade wearable devices, enabling large-scale screening initiatives [7].

This review aims to systematically evaluate the current applications of AI and ML in arrhythmia detection and pacemaker data analysis, focusing on the application gap of AI-augmented functions in mainstream commercial ECG systems and the innovative value of AI technology in pacemaker spike signal analysis, discuss the strengths and limitations of these technologies, examine the evidence supporting their clinical use, and explore future directions. The article will first provide an overview of the technical foundations, then delve into specific applications in arrhythmia identification and pacemaker data analytics, and finally address challenges related to clinical implementation, ethical considerations, and future prospects.

Despite the growing number of studies and reviews focusing on AI/ML applications in cardiac arrhythmia diagnosis, existing relevant systematic reviews still have obvious research deficiencies and perspective limitations, which fail to comprehensively cover the latest research progress and practical clinical pain points. First, most existing reviews focus narrowly on single-modal surface ECG AI diagnosis for common arrhythmias, while ignoring the unique clinical value of implantable pacemaker monitoring data. They lack systematic sorting and in-depth discussion on AI intelligent analysis of pacemaker-specific data, especially the fine-grained identification and clinical application of pacemaker spike signals that are easily neglected by conventional detection methods. Second, prior studies rarely focus on the real-world application status and existing bottlenecks of mainstream commercial ECG analysis systems (GE and Siemens), which dominate clinical diagnosis, resulting in disconnection between academic research and actual clinical practice. Third, existing literature often independently discusses technical application advantages, clinical transformation obstacles, and ethical regulatory issues in a scattered manner, without integrating the internal logical correlation between technological iteration, clinical verification defects, and ethical and institutional constraints, failing to form a systematic research framework. In addition, most reviews only enumerate technical achievements but lack targeted analysis of core restrictive factors such as data standardization inconsistencies and algorithm interpretability defects, as well as corresponding prospective solution paths.

Against this background, this review fills the above research gaps in the field. It takes the dual core of “arrhythmia intelligent diagnosis” and “pacemaker big data AI analysis” as the research perspective, integrates and summarizes the latest progress of AI-augmented mainstream clinical ECG systems and pacemaker spike signal analysis for the first time, systematically sorts out the technical iteration logic from traditional ML to deep learning in cardiac data analysis, centrally summarizes core clinical transformation challenges and ethical regulatory dilemmas, and puts forward targeted, hierarchical future research directions. This work aims to provide a comprehensive, practical, and forward-looking theoretical reference for the clinical popularization and standardized development of AI/ML technology in arrhythmia management and pacemaker precision therapy, highlighting the unique innovation and research necessity of this review.

## AI and ML Technology Basis

Before delving into specific applications, it is essential to clarify the relevant technical concepts.AI is a branch of computer science focused on developing systems capable of mimicking intelligent human behavior [8]. ML, a core subfield of AI, involves the development of algorithms that learn from data and improve through experience, identifying patterns and making predictions without being explicitly programmed for each task.

### Traditional ML methods

Traditional ML approaches encompass both supervised and unsupervised learning. Common supervised learning algorithms employed in cardiology include Support Vector Machines, Random Forest, k-Nearest Neighbors, and Logistic Regression [9]. These methods typically require manual feature extractions such as P-wave morphology, QRS complex width, and RR interval variability from ECG signals. For instance, Clifford et al successfully classified arrhythmias into five categories with an accuracy of 91.4% using an SVM classifier combined with ECG morphological and time-domain features [10]. Unsupervised learning methods, such as cluster analysis and principal component analysis, are suited for unlabeled data and can reveal underlying structures or anomalies in heart rhythm patterns [11]. Such techniques are particularly valuable for analyzing the vast amounts of unannotated data recorded by pacemakers.

### Deep learning approaches

Deep learning, an advanced branch of ML, utilizes artificial neural networks to automatically learn hierarchical feature representations from raw data [12]. In ECG signal analysis, several deep learning architectures have shown exceptional performance: Convolutional Neural Networks (CNNs) excel at capturing spatial features, making them particularly suitable for identifying morphological patterns in ECGs. Hannun et al developed a 13-layer CNN capable of automatically detecting 12 different types of arrhythmias from single-lead ECG recordings, achieving performance comparable to a panel of cardiology experts [13]. Recurrent Neural Networks, especially Long Short-Term Memory and Gated Recurrent Unit networks, are well-suited for ECG analysis due to their ability to capture temporal dependencies. Transfer learning, which leverages models pre-trained on large-scale datasets, has effectively addressed the challenge of limited labeled data in cardiology. For example, Raghunath et al fine-tuned a model pre-trained on 4.4 million ECGs to successfully predict long-term mortality risk in patients following myocardial infarction, attaining a C-statistic of 0.83 [14].

## Application of AI in Arrhythmia Identification

Accurate identification of cardiac arrhythmias is crucial for enabling timely intervention and improved patient outcomes. AI and ML technologies in this field have progressively transitioned from experimental research to clinical practice and are becoming increasingly integrated into real-world diagnostic workflows.

### Standard 12-lead ECG analysis

In the analysis of standard 12-lead ECGs, AI algorithms have demonstrated remarkable advantages. Ribeiro et al developed a deep neural network capable of identifying six common types of arrhythmias with an overall accuracy of 91%, specificity of 94%, and sensitivity of 87% [15]. More importantly, the algorithm was also able to detect subtle abnormalities that are often overlooked during human visual inspection. Going a step further, Attia et al trained an AI model that could identify a history of atrial fibrillation from seemingly normal ECGs, achieving an area under the curve (AUC) of 0.90, offering a promising tool for screening paroxysmal AF [16].

In terms of clinical commercial system application, mainstream ECG reading platforms represented by GE HealthCare and Siemens Healthineers dominate hospital automated ECG analysis workflows in the United States. Traditional versions of these systems entirely depend on rule-based programming algorithms, which can only realize basic identification of standard ECG waveforms and common arrhythmias, but have poor recognition ability for subtle arrhythmia changes, occult atrial fibrillation and non-typical waveform abnormalities, and are susceptible to baseline noise and artifact interference. In recent years, both manufacturers have launched independent AI-augmented ECG reading modules and completed preliminary Food and Drug Administration (FDA) certification and clinical verification, breaking through the limitations of traditional rule-based analysis.

GE HealthCare’s self-developed AI-ECG augmented framework integrates CNN models optimized for clinical ECG data, which can perform secondary intelligent analysis on standard 12-lead ECGs. This augmented system has achieved excellent performance in screening low ejection fraction, hypertrophic cardiomyopathy and occult arrhythmias, and can capture subclinical ECG feature changes that cannot be identified by traditional automated systems, effectively making up for the missed diagnosis defect of rule-based algorithms. Siemens Healthineers’ latest two-dimensional (2D) HeartAI intelligent module realizes automated high-precision quantification of cardiac electrical and structural parameters, optimizes the noise resistance and waveform feature extraction ability of ECG analysis, and significantly improves the accuracy of automated diagnosis of complex arrhythmias.

Despite the technical progress of AI-augmented modules in mainstream commercial systems, these AI functions are still optional auxiliary plug-ins rather than default built-in functions of clinical systems. Restricted by insufficient prospective clinical trial evidence, unified clinical application specifications and cost factors, AI-augmented ECG reading technology has not been widely popularized in routine clinical practice in the United States. At present, the vast majority of hospital ECG diagnosis still adopts the traditional mode of rule-based automated preliminary reading combined with physician manual review, and the full clinical transformation of AI-augmented commercial ECG systems still faces certain obstacles.

When it comes to classifying complex arrhythmias, AI algorithms have shown performance surpassing conventional methods. Research by Siontis et al revealed that a deep learning model achieved 96.7% accuracy in distinguishing wide-complex tachycardias (supraventricular versus ventricular), significantly outperforming both traditional ECG criteria and non-electrophysiologist cardiologists [17]. Additionally, the deep neural network developed by Hannun et al exceeded the average performance of cardiologists in classifying 10 common arrhythmias based on F1-score, suggesting that AI may have reached or even surpassed human expert-level performance in certain diagnostic tasks [13].

### Holter and wearable device data analysis

Continuous data from long-term ambulatory ECG monitors and wearable devices present unique opportunities for AI applications. Wasserlauf et al utilized a CNN to analyze 24-h Holter recordings, achieving notable success in predicting the progression from paroxysmal to persistent atrial fibrillation with an accuracy of 82% [18]. This predictive capability holds significant potential for guiding early clinical intervention.

In the realm of wearable technology, the Apple Heart Study led by Perez et al enrolled 419,297 participants and demonstrated the efficacy of a photoplethysmography (PPG)-based algorithm in screening for atrial fibrillation, reporting a positive predictive value of 84% [19]. Bumgarner et al further validated the accuracy of the Apple Watch ECG app in detecting AF, showing a sensitivity of 98% and specificity of 90% compared to 12-lead ECG [20]. These studies indicate that the integration of AI with consumer-grade devices holds promise for enabling large-scale arrhythmia screening. Moreover, AI algorithms exhibit distinct advantages in handling signal noise and artifacts commonly encountered in real-world settings.

### Prediction of occult arrhythmias

AI is capable of not only identifying existing arrhythmias but also predicting future cardiac events. Tison et al demonstrated that deep learning models can detect subtle changes in continuous ECG data to predict the onset of atrial fibrillation an average of 4 h in advance, with a sensitivity of 79% [21]. Similarly, Kwon et al showed that combining heart rate variability features with deep learning enables the prediction of cardiac arrest risk, offering a novel tool for identifying high-risk patients [22].

## Application of ML in Pacemaker Data Analysis

Modern pacemakers and implantable cardioverter-defibrillators (ICDs) have evolved into sophisticated monitoring devices capable of recording a wide range of electrophysiological parameters such as intracardiac electrograms, heart rate variability, thoracic impedance, and patient activity data. These rich and diverse datasets offer broad application potential for AI and ML technologies.

### Pacemaker recorded cardiac rhythm event analysis

Intracardiac electrograms recorded by pacemakers provide more direct information on cardiac electrical activity than surface ECGs. A CNN-based algorithm developed by Noseworthy et al successfully automated the classification of arrhythmic events detected by pacemakers using Intracardiac electrograms (EGM) data, achieving an accuracy of 95.2% and significantly reducing false alerts [23]. In another study, Boon et al demonstrated that an ML algorithm improved accuracy by 15% over conventional threshold-based methods in discriminating true atrial fibrillation from noise artifacts [24].

In addition to conventional intracardiac electrogram and rhythm event analysis, AI-augmented approaches have made important progress in the fine-grained analysis of pacemaker spike signals, which are core characteristic waveforms of paced ECGs and contain unique clinical diagnostic information. Pacemaker spikes are transient high-amplitude electrical pulses generated by pacing devices. Their morphological characteristics, amplitude changes, temporal distribution and matching degree with subsequent myocardial electrical activity can directly reflect the working state of the pacing device, myocardial pacing capture efficiency, lead contact stability and early device failure signs. Conventional analysis methods can only roughly identify the presence or absence of spikes, but cannot extract micro-features of spikes, and cannot effectively distinguish valid pacing spikes from electromagnetic noise, baseline artifacts and pseudo-spike signals, resulting in the loss of key diagnostic information.

Current AI research in this field mainly adopts CNN and hybrid CNN-LSTM deep learning architectures, which can automatically extract high-dimensional micro-features of pacemaker spikes from noisy ECG and intracardiac electrogram data, realize intelligent classification and quantitative analysis of spike signals. Relevant verification studies have shown that AI-augmented spike analysis algorithms can accurately identify atrial/ventricular pacing spikes, screen out abnormal spikes caused by lead displacement, battery aging and electromagnetic interference, and evaluate real-time pacing capture efficiency, with a recognition accuracy of more than 94%. Meanwhile, AI spike analysis can effectively reduce the false positive rate of pacemaker monitoring alarms caused by artifact interference and assist clinicians in early identification of subclinical pacing dysfunction and potential arrhythmia risks induced by abnormal pacing.

Nevertheless, the AI-augmented pacemaker spike analysis technology is still in the stage of clinical research and preliminary small-sample verification and has not yet been transformed into commercial embedded modules. The existing research has the limitations of single-center data, lack of unified spike feature labeling standards and insufficient external prospective validation. There is still a certain gap between the current technical level and large-scale routine clinical application, which is one of the important directions for future pacemaker data intelligent analysis research.

For the analysis of complex arrhythmic events, an ensemble learning approach integrating multiple features, such as onset pattern, rate stability, and morphological variability, achieved high accuracy (93.4%) in classifying supraventricular tachycardias. This precise classification is particularly valuable for avoiding unnecessary ICD therapies. Additionally, Toma et al developed a hybrid CNN-LSTM network capable of identifying early ventricular tachycardia patterns from continuous EGM recordings, offering a critical time window for preventive intervention [25].

### Predictive analysis of remote monitoring data

The continuous data generated by remote monitoring of pacemakers provides an ideal foundation for predictive analytics. In the IN-TIME study conducted by Hindricks et al [26], ML was applied to analyze remote monitoring data from ICDs, including arrhythmic events, lead impedance, and battery status, and successfully predicted heart failure decompensation. This approach reduced the detection time for clinical events from 30 days under conventional follow-up to an average of just 5.7 days [26]. Multiparameter fusion offers even greater potential. One study developed a multimodal learning framework that integrated EGM, heart rate variability, and patient physical activity data, improving accuracy by 13% in predicting adverse cardiovascular events compared to single-parameter models [27]. Furthermore, Cikes et al demonstrated that an ML model combining pacemaker data with clinical information could identify high-risk patient subgroups requiring closer monitoring, offering a novel approach to stratified management [28].

### Pacemaker parameter optimization

ML also demonstrates significant potential for the personalized optimization of pacemaker parameters. An ML-based adaptive pacing algorithm can dynamically adjust parameters according to a patient’s activity levels, leading to notable improvements in exercise tolerance and quality-of-life scores. For complex parameter optimization challenges, such as the coordinated adjustment of multiple parameters in cardiac resynchronization therapy, Crozier et al developed a system combining computational modeling and reinforcement learning to predict the hemodynamic effects of various parameter combinations. This system provides clinicians with personalized parameter recommendations [29]. Such a “digital twin” approach offers a novel paradigm for precision medicine in pacemaker therapy.

## Clinical Validation and Implementation Challenges

To avoid scattered and repetitive discussion of restrictive factors, this chapter centrally integrates and elaborates all core clinical transformation challenges of AI/ML technology in arrhythmia detection and pacemaker data analysis, including evidence-level defects, data standardization and quality bottlenecks, algorithm interpretability and clinical acceptance barriers, forming a systematic and hierarchical challenge system, and eliminating repeated descriptions in other chapters.

### Level of evidence for clinical validation

Despite the considerable promise of AI/ML applications in cardiology, the majority of current research remains in the retrospective validation phase, with a notable lack of prospective randomized controlled trials (RCTs) [30]. A systematic review by Briganti et al of AI studies in cardiology published between 2010 and 2020 revealed that only 4% reached level II evidence, with none attaining level I [31]. This evidence gap significantly hinders the broader clinical adoption of AI technologies. This problem is particularly prominent in the clinical transformation of AI-augmented mainstream ECG systems and pacemaker spike analysis technologies. The AI functional modules of GE and Siemens commercial systems lack large-scale multi-center prospective RCT verification of real-world clinical efficacy, and there is no unified evaluation standard for the clinical benefit of AI-augmented reading. Similarly, AI pacemaker spike analysis research is limited to retrospective data verification and small-sample prospective observation, lacking high-level evidence to support routine clinical application, which severely restricts its clinical popularization. Recently, several prospective studies have begun to address this shortfall. For instance, a prospective study by Perez et al evaluated the performance of a smartwatch-based atrial fibrillation detection algorithm among 25,000 participants, confirming its high accuracy in real-world settings [19]. Similarly, Steinhubl et al compared conventional care with AI-assisted remote monitoring for preventing heart failure readmissions and found that the latter reduced rehospitalization rates by 27% [32]. Despite these encouraging results, such studies still fall short of the rigorous standards required for large-scale RCTs.

### Data quality and standardization

Data quality and standardization represent fundamental challenges for AI applications in this field. Pacemaker data formats vary across manufacturers, lacking unified standards [33]. Furthermore, data noise in clinical settings remains a significant concern. Research by Hannun et al demonstrated that noise in ECG recordings can reduce the accuracy of AI algorithms from 95% to below 80% [13]. To address these issues, professional organizations such as the Heart Rhythm Society (HRS) and the European Society of Cardiology have begun developing data standards and quality control guidelines [3]. The ECG standardization framework proposed by Kligfield et al offers an essential foundation for AI algorithm development [34]. Additionally, emerging techniques like self-supervised learning are being explored to mitigate data quality challenges. For instance, one study developed a contrastive self-supervised framework capable of learning meaningful representations from unlabeled noisy data, thereby enhancing algorithmic robustness [35].

### Algorithm interpretability and clinical acceptance

The “black box” problem remains a significant barrier to the clinical adoption of AI. Physicians require understandable explanations of algorithmic decisions to build trust. Kwon et al addressed this using class activation mapping techniques to visualize the temporal and spectral features that CNNs focus on when identifying various arrhythmias [36].

Regarding clinical acceptance, a survey revealed that while 72% of cardiologists acknowledged the potential value of AI, only 35% were willing to rely on AI recommendations for critical decisions [37]. This discrepancy underscores the importance of improving algorithmic transparency and interpretability. Shah et al proposed a “human-AI collaboration” framework, positioning AI as a decision-support tool rather than a replacement. This approach has achieved higher acceptance among clinicians in clinical trials [38].

## Ethical and Regulatory Considerations

The rapid development of AI/ML technology also poses unique ethical and regulatory challenges that are particularly important in cardiology applications involving critical life safety. Different from the technical and clinical challenges in “Clinical Validation and Implementation Challenges”, this chapter focuses on institutional and social dimension risks, including data privacy security, algorithm fairness bias, and lagging regulatory systems, forming a complete set of restrictive factor systems together with clinical technical challenges.

### Data privacy and security

Remote monitoring systems for pacemakers and AI analysis platforms involve the transmission and storage of sensitive health data, making security a critical concern. Cohen et al note that pacemaker data could potentially be used for individual identification and health inference, raising serious privacy issues [39]. Furthermore, research by Kramer et al has exposed potential security vulnerabilities in pacemaker remote communication systems that could be exploited for unauthorized access or interference [40]. Regulatory bodies have begun addressing these challenges. The European Union’s (EU’s) General Data Protection Regulation classifies health data as a special category, mandating stricter protective measures. Similarly, the US FDA has issued guidelines on medical device cybersecurity, placing particular emphasis on risk management for remotely accessible devices. Within the industry, Slotwiner et al have proposed a comprehensive data governance framework for cardiac implantable electronic devices. This includes multi-layered security measures such as encrypted transmission, de-identification procedures, and strict access controls [3].

### Algorithm bias and equity

The fairness of AI algorithms presents another critical consideration. Due to demographic imbalances in training data, algorithms may exhibit systematic biases [41]. A study by Char et al found that an ECG analysis algorithm trained predominantly on data from White individuals showed an 11% reduction in accuracy when applied to an African American population [42]. Similarly, Yao et al reported that an atrial fibrillation detection algorithm trained on US and European data demonstrated significantly lower specificity in Asian cohorts [43]. To address these issues, researchers and regulatory bodies are taking various measures. The US FDA now requires developers of AI/ML-based medical devices to provide performance data across different demographic subgroups.

### Evolution of regulatory frameworks

Regulatory frameworks are rapidly adapting to accommodate the unique characteristics of AI/ML technologies. In 2021, the US FDA released the “Artificial Intelligence/Machine Learning Action Plan,” which outlines a novel regulatory pathway for “continuous learning” systems. This framework would permit certain AI/ML systems to update autonomously within predefined boundaries while maintaining regulatory oversight. Similarly, the European Medical Device Regulation has introduced new classification and evaluation requirements for software as a medical device [44]. In practical terms, Shah et al recommend adopting a “Predetermined Change Control Plan” model, where potential algorithm modifications and corresponding performance monitoring metrics are predefined [45]. This approach strikes a balance between fostering innovation and ensuring safety. Industry organizations such as the American Medical Association have also issued ethical guidelines for AI in healthcare, emphasizing transparency, accountability, and clinical validity [46].

## Future Directions

In response to the systematic core challenges summarized in in the preceding two sections (Clinical Validation and Implementation Challenges and Data Privacy and Security), this chapter abandons repetitive problem description, and proposes targeted, one-to-one corresponding future development strategies and technical optimization directions, forming a rigorous logical closed loop of “challenge analysis-solution optimization”, effectively avoiding full-text content redundancy and improving logical compactness.

### Multimodality fusion and holographic heart health

Future research will increasingly focus on multi-source data integration to construct a “holistic” cardiac health profile [47]. A study by Wang et al demonstrated that a multimodal deep learning model combining ECG, pacemaker data, and electronic health records achieved a 12% higher accuracy compared to models using a single data source [48]. Tison et al proposed the concept of a “cardiac digital twin,” which integrates multi-source real-time data with personalized computational models, offering a new paradigm for precise prediction and intervention [7]. Future multimodal fusion research will also focus on the deep fusion of pacemaker spike micro-features, intracardiac electrogram signals and surface ECG data, relying on AI augmentation to fully mine the clinical value of spike signals, and build a more comprehensive intelligent evaluation system for pacing function and arrhythmia risk. Another significant trend involves the synergy between wearable and implantable devices. Research by Bumgarner et al showed that combining pacemaker recordings with smartwatch data enables more comprehensive monitoring of arrhythmic patterns and their correlation with patient symptoms [20]. This “inside-out” approach holds promise for continuous and all-round physiological monitoring.

### Federal learning and privacy protection technology

Balancing data privacy with research demands will be achieved through advanced computational techniques. Federated learning enables algorithms to learn from multiple institutions without sharing raw patient data [49]. Teo et al demonstrated that a federated learning framework could train a high-performance arrhythmia detection model using ECG data from 10 hospitals while preserving patient privacy [50]. Furthermore, emerging privacy-preserving technologies such as homomorphic encryption and differential privacy are expected to play a crucial role in enabling secure cardiac data analysis [51].

### Adaptive learning and personalized prediction

Future algorithms will place greater emphasis on personalization and adaptability. A personalized deep learning framework developed by Clifford et al can dynamically adjust model parameters based on individual patient ECG characteristics, achieving a 7% improvement in accuracy compared to generic models [10]. The application of reinforcement learning in optimizing pacemaker parameters is also expected to advance. Continuous learning capability represents another key direction. Kwon et al proposed an “adaptive rhythm monitoring” framework capable of learning incrementally from new data, enabling algorithm performance to improve rather than degrade over time [52]. This capability is particularly crucial for the long-term optimization of implantable devices.

## Conclusions

AI and ML technologies demonstrate significant potential in arrhythmia detection and pacemaker data analysis. Deep learning algorithms have achieved or surpassed expert-level performance in certain arrhythmia recognition tasks, while ML methods have yielded encouraging results in predictive analytics for pacemaker data. These technologies are transitioning from proof-of-concept stages to clinical practice, promising improved diagnostic accuracy, accelerated treatment decisions, and personalized interventions.

Nevertheless, multiple challenges persist in this domain, including data standardization, algorithm explainability, clinical validation, and ethical and regulatory considerations. Addressing these challenges requires multidisciplinary collaboration among cardiologists, data scientists, ethicists, and regulatory authorities. As technologies mature, increasingly transparent, reliable, and personalized AI systems have the potential to become powerful adjuncts to clinical decision-making in cardiology, ultimately improving patient outcomes and quality of life.

Future research should prioritize conducting large-scale prospective clinical trials to establish evidence-based foundations for AI applications; developing more transparent and explainable algorithms to enhance clinical acceptance; exploring advanced technologies such as multimodal integration and federated learning to improve algorithmic performance while preserving privacy; and designing adaptive learning systems to achieve true precision cardiology. By addressing these key issues, AI/ML technologies can potentially assume a more central role in future arrhythmia management and cardiac device therapy.

## Data Availability

The data used to support the findings of this study are available from the corresponding author upon request.

## Abbreviations

AI: artificial intelligence
ML: machine learning
